# Genome-wide association study and expression of candidate genes for Fe and Zn concentration in sorghum grains

**DOI:** 10.1038/s41598-024-63308-0

**Published:** 2024-06-03

**Authors:** Niranjan Ravindra Thakur, Sunita Gorthy, AnilKumar Vemula, Damaris A. Odeny, Pradeep Ruperao, Pramod Ramchandra Sargar, Shivaji Pandurang Mehtre, Hirakant V. Kalpande, Ephrem Habyarimana

**Affiliations:** 1 International Crops Research Institute for the Semi-Arid Tropics, Patancheru, Telangana India; 2 Vasantrao Naik Marathwada Agriculture University, Parbhani, Maharashtra India

**Keywords:** Gene expression profiling, Genome-wide association studies

## Abstract

Sorghum germplasm showed grain Fe and Zn genetic variability, but a few varieties were biofortified with these minerals. This work contributes to narrowing this gap. Fe and Zn concentrations along with 55,068 high-quality GBS SNP data from 140 sorghum accessions were used in this study. Both micronutrients exhibited good variability with respective ranges of 22.09–52.55 ppm and 17.92–43.16 ppm. Significant marker-trait associations were identified on chromosomes 1, 3, and 5. Two major effect SNPs (S01_72265728 and S05_58213541) explained 35% and 32% of Fe and Zn phenotypic variance, respectively. The SNP S01_72265728 was identified in the cytochrome P450 gene and showed a positive effect on Fe accumulation in the kernel, while S05_58213541 was intergenic near Sobic.005G134800 (zinc-binding ribosomal protein) and showed negative effect on Zn. Tissue-specific in silico expression analysis resulted in higher levels of Sobic.003G350800 gene product in several tissues such as leaf, root, flower, panicle, and stem. Sobic.005G188300 and Sobic.001G463800 were expressed moderately at grain maturity and anthesis in leaf, root, panicle, and seed tissues. The candidate genes expressed in leaves, stems, and grains will be targeted to improve grain and stover quality. The haplotypes identified will be useful in forward genetics breeding.

## Introduction

After a decades-long fall and five years of stability since 2014, the global Prevalence of Undernourishment (PoU) spiked during 2019 and 2020 due to the COVID-19 pandemic^1^. In 2020, 21% of the population in Africa was hungry^1^. Asia's enormous population houses 54% of the world's hungry^1^. According to the World Health Organization^2–4^, 149.2 million children under five were stunted, with diet-related deaths amounting to 45%, whereas 462 million adults were underweighting in 2014. African region tops this critical situation with 56.2 million children, followed by 49.8 million in South-East Asia, and 22.9 million children in the Eastern Mediterranean region^5^. Malnutrition is the inadequate consumption of micronutrients like iodine, vitamin A, iron, and zinc which affects worldwide public health^2,6^. Therefore, inadequate nutrition threatens the health and development of populations worldwide, especially children and pregnant women in countries with lower income.

The importance of iron (Fe) in human food cannot be overemphasized. Iron deficiency anemia is a common type of anemia described as blood lacking adequate healthy red blood cells carrying oxygen to the body's tissues^7–9^. Anemia decreases physical and mental capabilities but is rarely discovered, and when worsens it results in death. Anemia during pregnancy can impede growth and development due to preterm delivery, low-birth-weight infants, maternal death, and reduced iron storage. In 2019, 29.9% of reproductive-age women (nearly half a billion women aged 15–49), 36.5% of pregnant women, and 39.8% of 6–59-month-olds (269 million children) had anemia^10^.

On the other hand, zinc, one of the most abundant trace elements, is the most widely distributed trace element in the body after iron. Zinc has a structural role in over 2500 transcription factors, is necessary for the activity of over 300 proteins structure and enzymes activity, and controls thousands of genes and gene expressions^11,12^. Zinc is important in DNA synthesis, cell proliferation, protein synthesis, wound healing, and immune system support. It considerably impacts macrophages, neutrophils, and other complementary activity at the microcellular level^13–15^. Two billion people are still at risk of zinc deficiency, with infants, toddlers, and pregnant and lactating women being the most vulnerable due to their higher zinc requirements^16,17^. Zinc must be consumed or supplemented because the body cannot store it; its insufficiency is a global health issue, especially in developing nations like India, Pakistan, Ethiopia, Indonesia, and Vietnam^18^.

External nutrient supplementation and fortification are the most widely used methods to counter micronutrient malnutrition. Biofortified foods can also be produced through plant breeding and/or agronomic practices designed to increase the density of vitamins and minerals in the crop of interest. It is now acknowledged that developing high-nutrient cultivars is cheap, and making it affordable for low-income populations is a promising strategy to reduce micronutrient malnutrition.

Sorghum is an ideal crop under current and future climate change scenarios because it generates a good net return with low input and can feed a larger population, especially in semi-arid ecologies where wheat (*Triticum aestivum* L.), maize (*Zea mays* L.), and rice (*Oryza sativa*) cannot be produced sustainably due to climate adversities, particularly high temperatures and increased drought stress^19,20^. Sorghum is a staple food for more than 500 million people in Africa and Asia^21^. The estimated global sorghum consumption is expected to reach 26.5 million metric tons by the year 2026. Nigeria emerged as the leading consumer of sorghum in 2021, consuming a staggering 5.7 million metric tons. India, China, and Ethiopia were next, with 3.9, 3.7, and 3.5 million metric tons, respectively^22^. Due to its low glycemic index and high antioxidant content, sorghum grain is a popular gluten-free and celiac-safe diet^23^, and its consumption is expected to increase in developed countries driven by not only its life-promoting properties but also by its cultivation as a substitute of major cereals in those parts of the world. Biofortifying sorghum using conventional plant breeding and molecular technologies approaches can improve sorghum as a nutrient-dense and climate-resilient crop.

Previous studies on grain Fe and Zn concentrations in sorghum found significant genetic variability and strong heritability^24,25^. Madhusudhana et al.^26^ recently observed a highly significant G × E for Fe, but not for Zn. A significant G × E indicates that a genotype can perform well in particular environments but not others. Data from several trials showed high broad sense heritability for sorghum grain Fe (> 85%) and Zn concentrations (> 82%)^27^. Kumar et al.^23^ concluded that additive gene action controls Zn, and therefore high Zn lines can be developed by increasing the frequency of favorable alleles through intercrosses and other population improvement strategies. Additive and non-additive gene actions were found to regulate Fe concentration^23^, implying that this trait can be improved through heterosis breeding on the one hand, and crossing and selecting superior progeny, on the other. The highly significant and positive correlation between general combining ability (GCA) and line performance per se suggested that parental genotype performance per se can predict the grain Fe and Zn concentrations in hybrids and breeding lines^23^.

Although sorghum germplasm has shown large variability and genetic heritability for iron and zinc content^25^, only a few Fe and Zn biofortified varieties have been released thus far^28^. Indeed, the first India’s biofortified sorghum was released only two years ago, in 2022^29^. This work is, therefore, a contribution to the identification of major quantitative traits loci (QTLs) and genes governing Fe and Zn biofortification for use in breeding e.g., through introgressions in a sustained marker-assisted selection to create new biofortified varieties and improve the nutritional quality of the existing cultivars.

Recently, biparental mapping populations were used to identify QTLs for Fe and Zn in sorghum^24^. However, the QTLs found using this method were of low resolution. In contrast, linkage disequilibrium (LD)-based association mapping (Genome-Wide Association Study) can boost mapping resolution by representing a more diverse gene pool and accounting for past meiotic events. This technique was used in various previous research works to find markers non-randomly associated with the phenotype of interest in a broader process of forward genetics and breeding. Girma et al.^30^, Enyew et al.^31^, Wondimu et al.^32^ carried out GWAS on sorghum agro-morphological traits, while other GWAS were successfully conducted on nutritional traits such as polyphenol^33,34^, protein, fat, and starch^35^, mineral traits^36^, and biomass related traits^37^. Several genomic regions governing grain Fe and Zn have already been identified in other cereals such as Pearl Millet^38,39^, Wheat^40,41^, Maize^42^, Barley^43^, and Rice^44^. However, no reports on identifying SNPs and genes controlling grain Fe and Zn in sorghum are available. This work was therefore undertaken to close this gap; it is the first to report the marker traits associations (MTAs) for grain Fe and Zn in sorghum using SNPs and minicore lines. We aimed at identifying SNPs associated with grain Fe and Zn concentration in the sorghum kernel, mining underlying candidate genes using a part of the ICRISAT’s minicore set of sorghum collection, and implementing several GWAS models. ICRISAT’s genebank is the repository of the largest sorghum germplasm collection i.e., over 42,880 accessions from over 94 countries worldwide (http://genebank.icrisat.org/). The mini core, a gateway to the germplasm, is highly genetically diverse and is an efficient option for carrying out association mapping and allele mining for traits of interest^45^. The outcome of this work is, therefore, expected to benefit the scientific community across the globe.

## Materials and methods

### Multi-environment field trials and plant materials

The experimental materials for the current study consisted of 140 diverse germplasm accessions representing a part of a sorghum minicore maintained at the International Crops Research for the Semi-Arid Tropics (ICRISAT, Patancheru), from 58 different countries (Supplementary Table S1). These germplasm accessions were evaluated for grain Fe and Zn content (ppm) in post-rainy seasons of two consecutive years across two locations. The seasons included post rainy 2020 (PR 2020) and post rainy 2021 (PR 2021), while the locations were the International Crops Research Institute for the Semi-Arid Tropics (ICRISAT, Patancheru) (17.53°N, 78.27°E) located at an altitude of 545 m above mean sea level and Vasantrao Naik Marathwada Krishi Vidyapeeth (VNMKV, Parbhani) (18.45°N, 76.13°E) located at an altitude of 357 m above mean sea level. Soil testing analysis using the DTPA method for the experiment fields at Parbhani was done at the Department of Soil Science, COA, Parbhani, while, for ICRISAT fields it was done at Charles Renard Analytical Laboratory, ICRISAT, Patancheru. The available Fe and Zn content in our research field was beyond the minimum required levels (4.50 to 6.50 mg kg^−1^ Fe and 0.6 to 0.9 mg kg^−1^ Zn)^46^ and ample for normal growth and development of plants. Soil type at Parbhani was deep and black whereas, at ICRISAT it was shallow and light black. Irrigation was provided at each important crop stages to grow healthy plants including the grains development stage. The experiments were conducted in the post-rainy season to ensure the good quality of the seed. The experimental design was alpha lattice with two replications. Each germplasm was grown in a single row of 4 m long at both locations with inter and intra-row spacing of 60 × 15 cm. Standard agronomic practices were followed for successful crop development in each season.

### Phenotyping and estimation of Fe and Zn concentration in the grains

The panicles were harvested to measure the Fe and Zn content from the grains as they reached the physiological maturity stage. The panicles from five random plants from each plot were selfed before the flowering stage to obtain pure seed. Upon maturity, these panicles were harvested and stored separately in a dry cloth bag to produce clean grain samples for micronutrient analysis.

The harvested panicles underwent a 7-day sun drying to achieve a post-harvest grain moisture level of 10–12%, essential for mitigating fungal infections. Threshing was meticulously conducted in cloth bags to prevent sample contamination by extraneous particles. Subsequently, the seeds underwent cleaning to eliminate glumes, anthers, dust, and other contaminants. A composite seed sample weighing approximately 30 g was then collected for each plot. Analysis of grain Fe and Zn content was performed using X-ray fluorescence spectrometry (XRF) (Make: Hitachi High-Tech, Japan; model: X-Supreme8000), a calibrated, efficient, non-destructive, and cost-effective analytical method.

### Statistical analyses

A combined analysis of variance (ANOVA) was performed to assess the main and interaction effects of Environment (E) and Genotype (G), considering E, G, and replications as random effects. The individual variance of environments was modelled using the Residual Maximum Likelihood (REML) procedure using SAS Mixed procedure using SAS v9.4^47^. Best Linear Unbiased Predictors (BLUPs) were estimated using below equation:

$${Y}_{ijkl}=M+{E}_{i}+{R(E)}_{j(i)}+{G}_{k}+{GE}_{jk}+{e}_{ijkl}$$

where ${Y}_{ijkl}, M, {E}_{i}, {R(E)}_{j(i)}, {G}_{k}, {GE}_{jk},\text{ and }{e}_{ijkl}$, respectively, stand for the measurement on plot $l$ in environment $i$, block $j$, containing genotype $k$, the overall mean of all plots in all environments, the effect of environment (trial) $i$, the effect of replicate $j$ within environment $i$, the effect of genotype $k$, the interaction of genotype $k$ with environment $i$, the plot residual.

The heritability was estimated as repeatability^48^ using the formula:

$$H^{2} = \frac{{\sigma_{g}^{2} }}{{\sigma_{g}^{2} + \left( {{\raise0.7ex\hbox{${\sigma_{gxe}^{2} }$} \!\mathord{\left/ {\vphantom {{\sigma_{gxe}^{2} } E}}\right.\kern-0pt} \!\lower0.7ex\hbox{$E$}}} \right) + \left( {{\raise0.7ex\hbox{${\sigma_{e}^{2} }$} \!\mathord{\left/ {\vphantom {{\sigma_{e}^{2} } {r*E}}}\right.\kern-0pt} \!\lower0.7ex\hbox{${r*E}$}}} \right)}}$$

where ${\sigma }_{g}^{2}, {\sigma }_{gxe}^{2}, and {\sigma }_{e}^{2}$ are genetic variance component, genotype × environment variance component and residual variance respectively; $E$ and $r$ are the number of environments and replications, respectively. The four best combinations of environments were selected for phenotypic data processing in this study, based upon the relatively high repeatability (Table 1) from the combined analysis of variance. All combinations of the environments are presented in Supplementary Table S2.

**Table 1:** Repeatability-based combinations of environments to generate BLUPs for downstream GWAS analyses.

Combination No	Environments*				Repeatability (%)	
	ICRISAT_20	ICRISAT_21	Parbhani_20	Parbhani_21	Fe	Zn
1	✓	✓	✓	✓	0.46	0.66
2		✓	✓	✓	0.41	0.57
3	✓		✓		0.45	0.64
4			✓	✓	0.56	0.74

*ICRISAT_20—post-rainy season of the year 2020 at International Crops Research Institute for the Semi-Arid Tropics, Patancheru; ICRISAT_21—post-rainy season of the year 2021 at International Crops Research Institute for the Semi-Arid Tropics, Patancheru; Parbhani_20—post-rainy season of the year 2020 at Vasantrao Naik Marathwada Agriculture University, Parbhani; Parbhani_21—post-rainy season of the year 2021 at Vasantrao Naik Marathwada Agriculture University, Parbhani; Fe—iron; Zn—zinc.

### Isolation of DNA and genotyping

Genomic DNA from the single plant of sorghum minicore samples was isolated from 30 days old seedlings using the QIAGEN DNAeasy 96 plant kit. Purity and quantity of the extracted DNA was determined using gel electrophoresis and a Qubit 2.0 Fluorometer (Life Technologies, Carlsbad, CA) respectively and finally diluted to 30 ng/µl. Genotype-by-sequencing (GBS) libraries were prepared using the restriction enzyme ApeK1 according to Elshire et al.^49^. SNPs were called using the TASSEL v5.2 GBS pipeline^50^ against the *S. bicolor* BTx623 reference genome v3.1.1 (www.phytozome.net)^51^ using Bowtie v2.5.1^52^ with default parameters. Raw SNPs were called from the 140 genotypes and subsequently filtered to retain bi-allelic markers with < 40% missing data as suggested in Ali et al.^53^, maximum of 20% of heterozygosity and a minimum of 5% minor allele frequency (MAF). The retained high-quality SNPs were used in this work for genetic diversity, population structure and marker-traits association analyses (MTAs). The MTAs were performed using R GAPIT 3 pipeline^54^.

### Population structure

The high-quality SNPs were used to determine the genetic distance between the sorghum accessions using R “amap” and “labdsv” packages. The phylogeny analysis was performed with the Euclidean method with 1000 bootstrap replications with R "ape" package^55^ and the neighbor-joining tree was visualized in iTOL tree viewer^56^. The Principal Coordinates Analysis (PCo) between the accessions was measured with R "labdsv" package (https://CRAN.R-project.org/package=labdsv). The admixture analysis was performed with ADMIXTURE 1.3.0^57^ with expected K in the range of 2 to 15 and the K with the lowest cross-validation error considered as optimal sub-populations. The genome-wide Linkage Disequilibrium (LD) was generated using the r^2^ values calculated with TASSEL v5.2^50^.

### Association mapping

We performed GWAS with multiple models viz., MLMM, SUPER, BLINK and FarmCPU. Manhattan and Quantile–Quantile (Q–Q) plots were visualized in RStudio^58^ using GAPIT 3 package^54^. The spurious associations in GWAS were corrected using Bonferroni Correction (5% level of significance)^59^ and significant MTAs, corresponding to putative QTLs for the studied traits, were determined by the *P-value* less than 0.05/m, with m being the number of markers^60^. In the present investigation, Bonferroni correction value is calculated at 9.07 × 10^–5^. Further, the percentage of phenotypic variance explained (PVE) by all significant SNPs was generated either by GAPIT 3 built-in algorithms or calculated in RStudio as the squared correlation between the phenotype (BLUPs) and genotype of the SNP accounting for the above-described populations of environments. The Linux bash and *in-house* developed script was used to find the haplotype blocks and to identify the promising germplasms exhibiting high Fe and Zn content from the sorghum population.

### Candidate gene identification and in silico gene expression analysis

Single nucleotide polymorphisms (SNPs) explaining more than 7.5% of the phenotypic variance of their associated traits were identified and their genomic regions were further analyzed in the process of functional GWAS and candidate gene identification. To perform functional GWAS, an interval of 51.77 Kb upstream and downstream the SNP position was considered, based on a genome-wide linkage disequilibrium (LD) decay cut-off at r^2^ = 0.1. Annotation details for genes and respective Gene Ontologies (GO) within each region were retrieved using the Phytomine interface implemented in Phytozome^61^.

JGI Plant Gene Atlas (https://plantgeneatlas.jgi.doe.gov/)^62^ was used to check the tissue-specific expression of candidate genes and to access the expression data for 36 tissues at juvenile, vegetative, and reproductive stages. Gene expression was in the unit of Fragments per kilobase of transcripts per million mapped reads (FPKM). The expression heatmap was generated using TBtools II^63^.

### Ethics statement

This research does not involve the ethics of human and animal experiments.

### Institutional, national, and international guidelines and legislation statement

The seeds of sorghum used in this work were collected from ICRISAT’s Genebank (http://genebank.icrisat.org/). The authors confirm that all methods applied in this work were performed in accordance with the relevant guidelines/regulations/legislation.

## Results

### Statistical analyses

The repeatability i.e., test–retest reliability, resulted relatively highest (56%) for Fe in combination 4, followed by combinations 1, 3 and 2 with 46, 45 and 41%, respectively. Similarly for Zn the highest repeatability values (74%) were observed in combination 4 followed by combinations 1, 3 and 2 with 66, 64 and 57%, respectively, as shown in Table 1.

**Figure 1:**
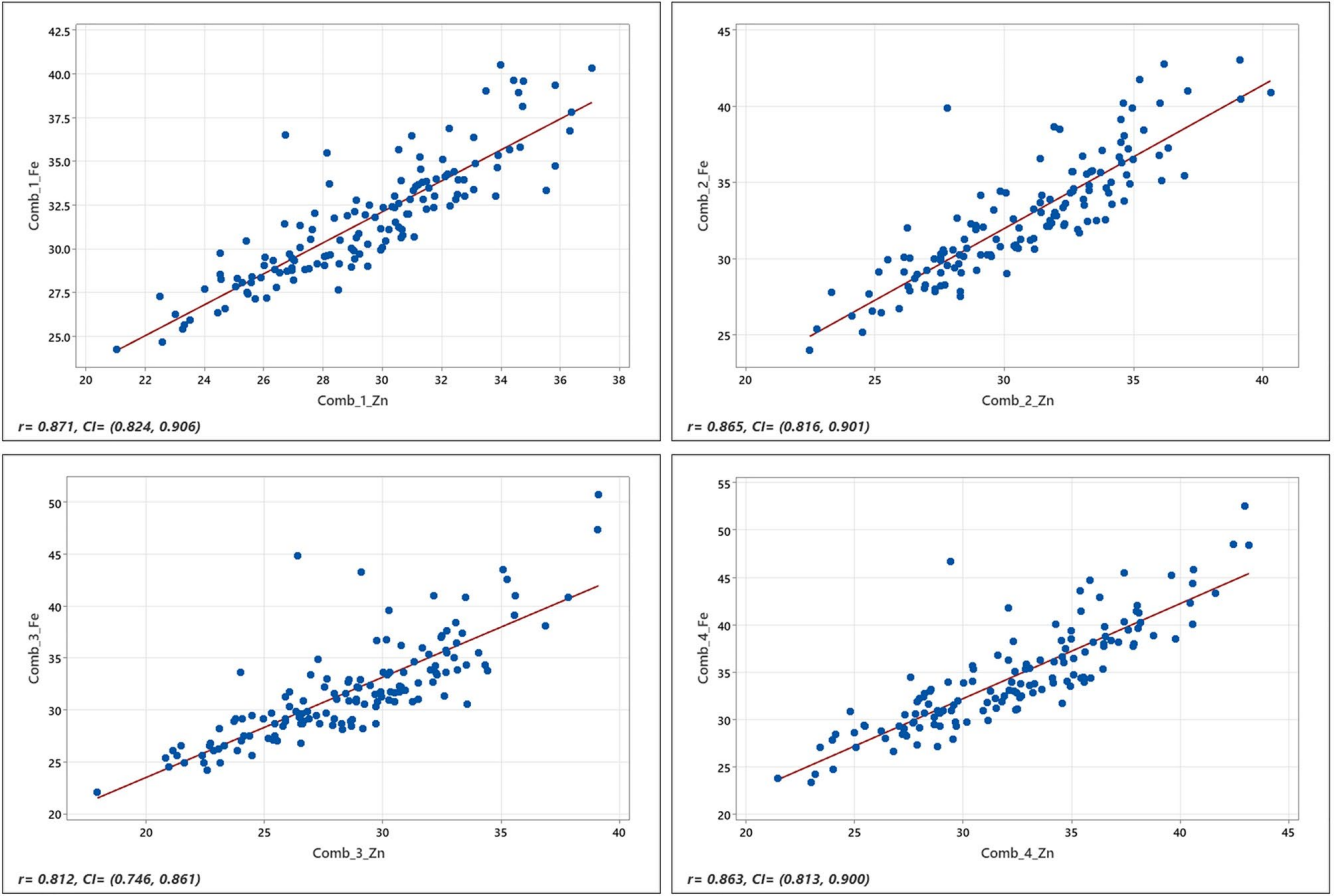
Relationship (Pearson correlation coefficient, r) between grain iron and zinc across four different combinations of environments with a confidence interval (CI) of 95%. Comb_1, _2, _3, _4, respectively, environments combination 1, 2, 3, and 4.

**Figure 2:**
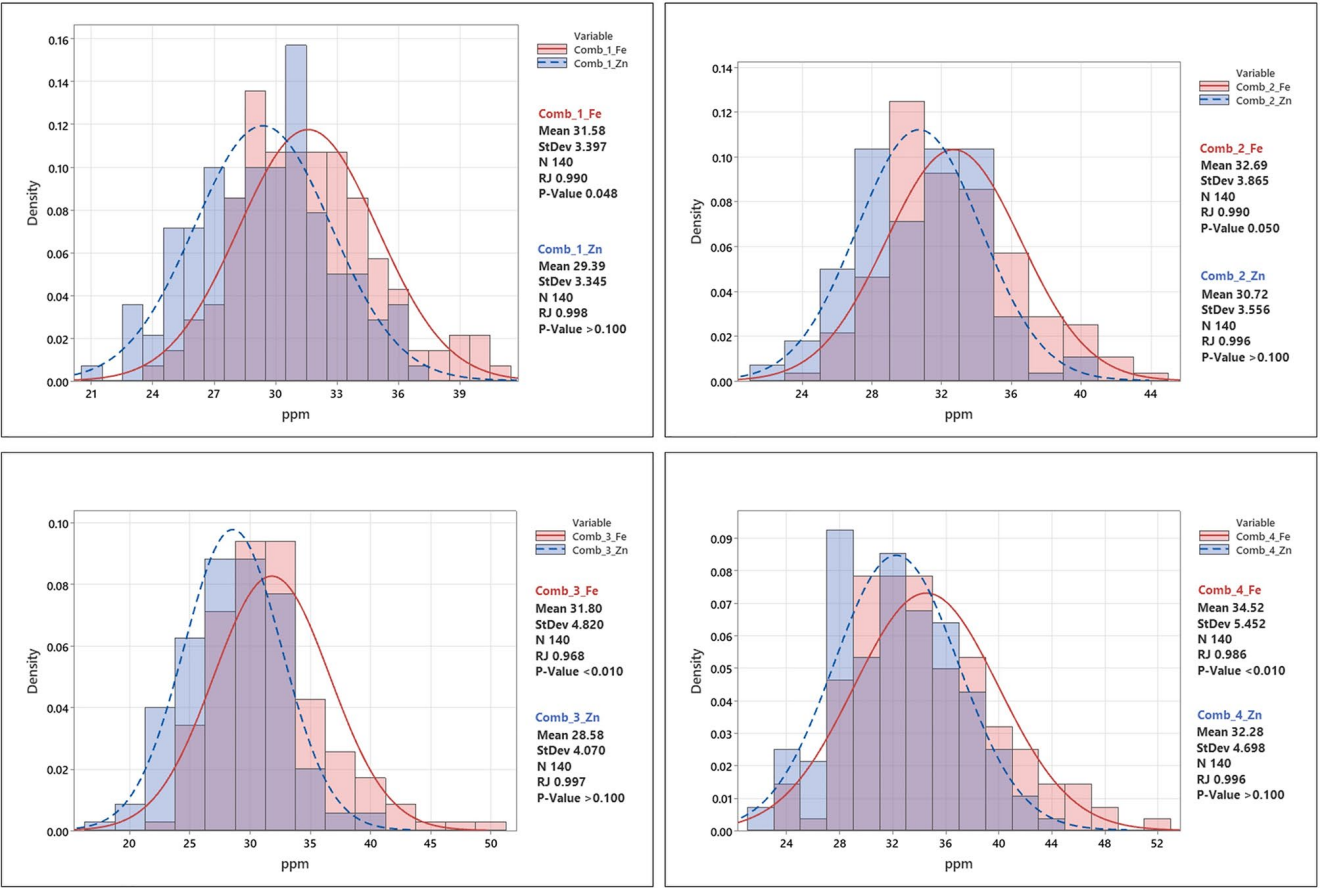
Graphical representation of grain iron and zinc frequency distributions for all four combinations. Comb_1, _2, _3, _4, respectively, environments combination 1, 2, 3, and 4. Red color bars indicate the distribution of grain iron and blue color bars indicate the grain zinc distribution. The distribution of population is normal in all four combinations as described in Shapiro–Wilk test for normality in frequentist statistics. The Ryan-Joiner (RJ) statistic also confirms that phenotypic data follow a normal distribution.

The combined ANOVA showed significant differences among the genotypes and a significant genotype × environment for both Fe and Zn traits (Table 2). As depicted in Table 2 and in Fig. 1, the concentration of Fe and Zn varied over a wide range, and the correlation between the two micronutrients was high (r > 0.80). The frequency distributions of the phenotypic data followed a normal distribution, as suggested by Shapiro—Wilks normality test^64^; and Ryan-Joiner statistics^65^ (Fig. 2).

**Table 2:** ANOVA for variance components for both Fe and Zn traits for four combinations.

Traits	Variance	Comb 1	Comb 2	Comb 3	Comb 4
Fe	Env	11.58 ***	10.18 **	24.601 **	1.08
	Env.Rep	0.24 **	0.24 *	0.097	0
	Gen	6.37 ***	7.4 ***	11.809 ***	19.03 ***
	Env.Gen	25.25 ***	27.37 ***	26.147 ***	25.85 ***
	Mean (ppm)	31.58	32.68	31.79	34.52
	Range (ppm)	24.26–40.5	24.01–43.03	22.09–50.7	23.37–52.55
	CV (%)	9.29	9.17	7.96	8.13
Zn	Env	11.74 ***	7.059 **	20.136 *	20.136
	Env.Rep	0.194 *	0.007	0.281 *	0.281
	Gen	8.669 ***	8.668 ***	12.484 ***	12.484 ***
	Env.Gen	14.069 ***	15.832 ***	10.954 ***	10.954 ***
	Mean (ppm)	29.39	30.72	28.58	32.27
	Range (ppm)	21.03–37.07	22.47–40.3	17.92–39.13	21.45–43.16
	CV (%)	9.56	9.07	8.38	7.32

*, **and *** denotes significant at the 5%, 1% and 0.1% probability level, respectively; Fe—Iron, Zn—Zinc, Comb—Combination of environments (as depicted in Table 1); Env—Environment; Env.Rep—Environment × Replications; Gen—Genotype; Env.Gen—Environment × Genotypes; ppm—parts per million (mg/kg); CV—coefficient of variation.

### Genetic diversity, population structure, and linkage disequilibrium (LD)

**Figure 3:**
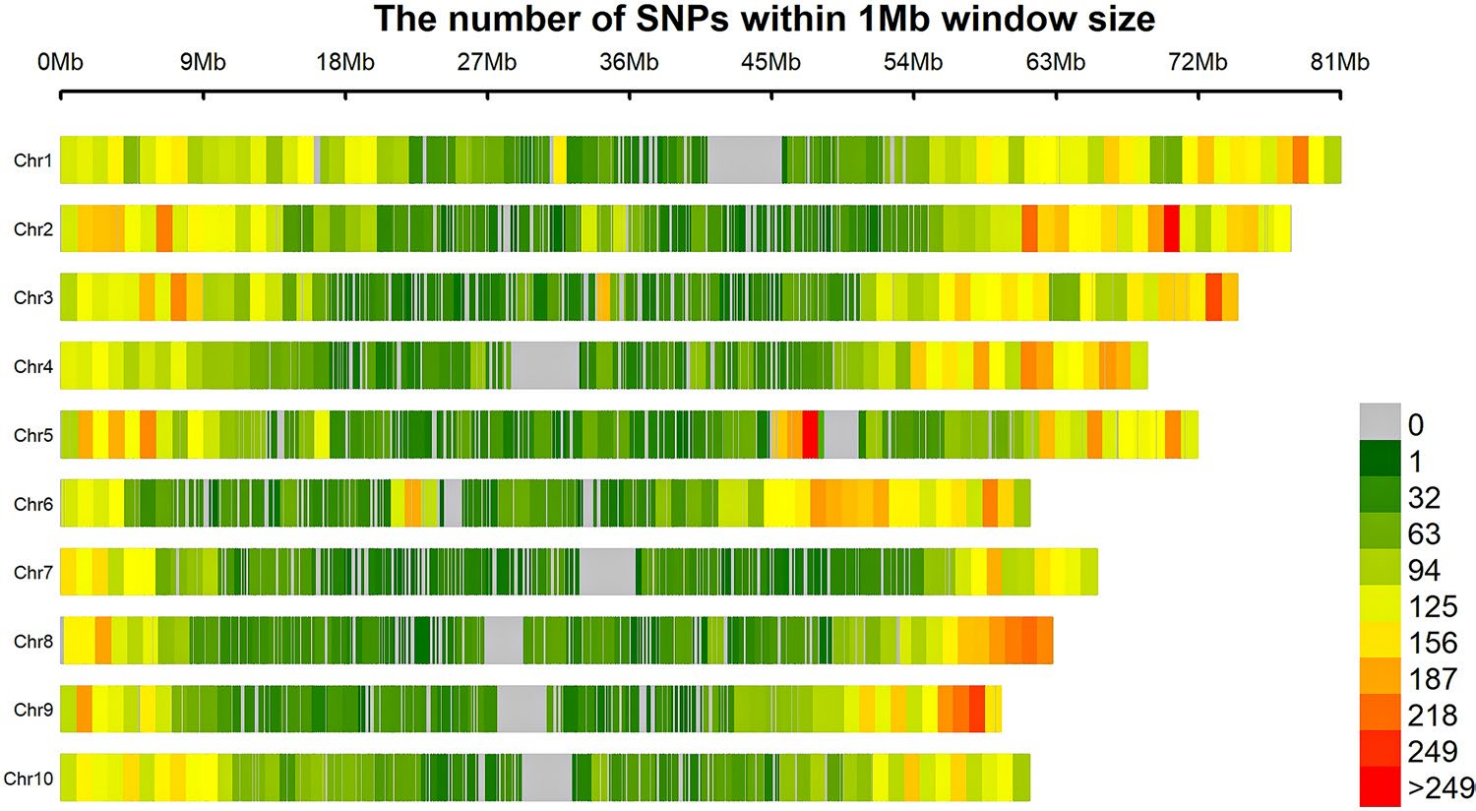
Distribution of SNP markers on ten chromosomes of the sorghum. Telomeric regions of the chromosomes are highly dense relative to the centromeric regions.

**Figure 4:**
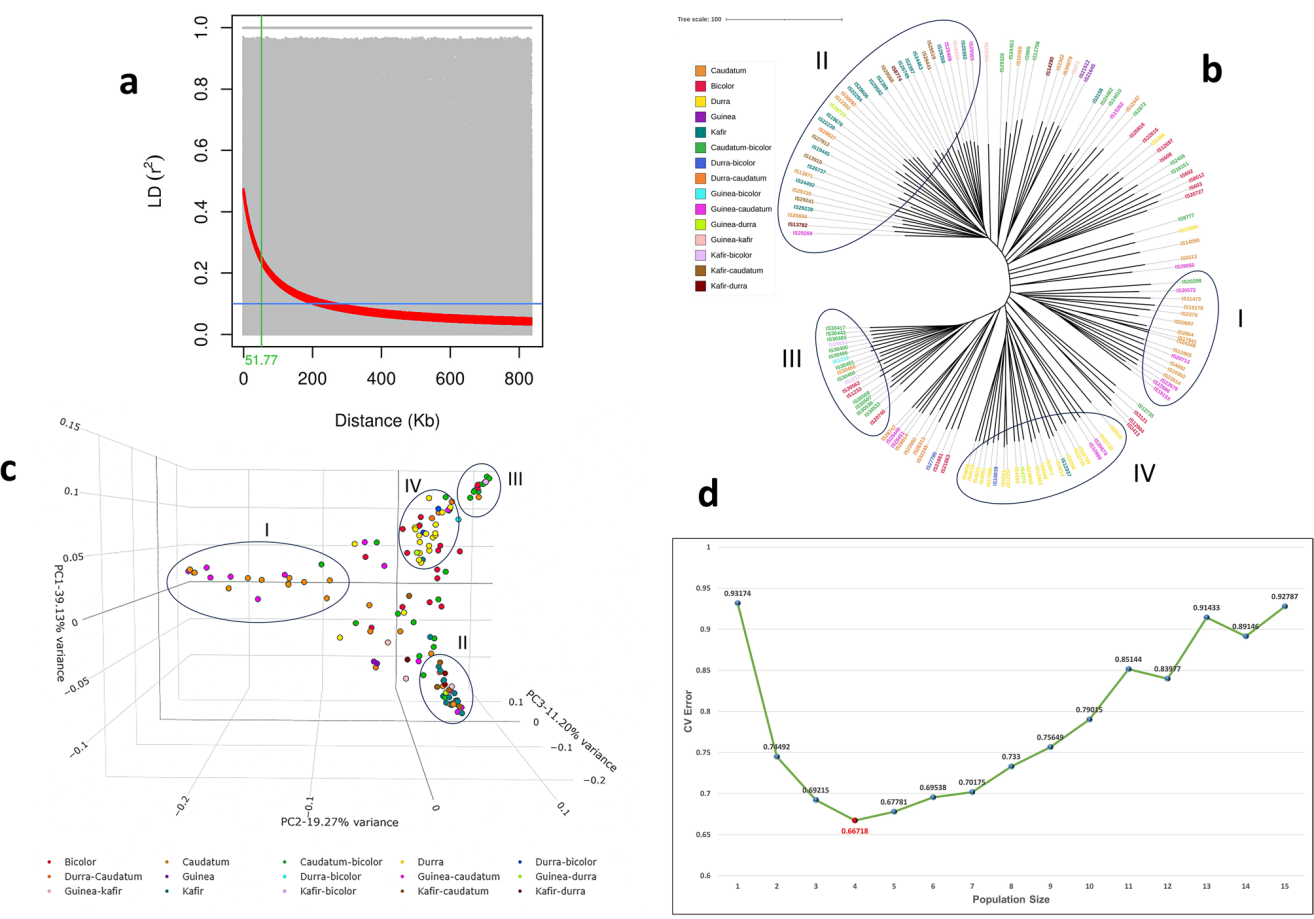
Informativeness of the markers and characterization of the structure of the genotypes used for GWAS. a. LD decay distance estimated at 51.77 Kb. b. A dendrogram showing the clustering of the genotypes used. Four clear clusters are observed with the rest appearing as admixtures. Cluster I—Caudatum and associated hybrids; Cluster 2—Kafir and associated hybrids; Cluster III—Caudatum-bicolor hybrids; Cluster IV—Durra genotypes. c. PCA showing informativeness of the markers and further confirming the clustering observed in the dendrogram. d. Optimum population size estimation confirms K = 4 as the point with the lowest CV error.

We retained 55,068 high quality SNPs that were well distributed across the genome (Fig. 3) with a density of ~ 5505 markers per chromosome (Chr) and around 80 markers per Mb region. Genomic regions with high marker density were observed on Chr 01 and Chr 02 with an average magnitude of 91 markers per Mb on both chromosomes. Chr 06 has averaged 86 markers per 1 Mb region with high-density regions between 47 and 52 Mb. Chr 08 and 09 also showed high-density regions between 57–63 Mb and 55–74 Mb, respectively. Linkage-disequilibrium decay (LD decay) is determined using the entire set of markers. The LD decay plot was plotted as LD (r^2^) against the distance in base pairs (bp). The overall LD decay across the genome estimated at 51.77 kb (Fig. 4a). The unweighted unrooted neighbor-joining tree (Fig. 4b) used to depict the phylogenetic diversity showed that the genotypes clustered based on the races, but also revealed a significant level of admixtures (Fig. 4b). A similar clustering was confirmed using the PCA with the first 3 PCs explaining 39.13%, 19.27% and 11.20% genetic variation (GVE), respectively (Fig. 4c), which gives a total of 70% GVE. The optimum population size estimation using the ADMIXTURE model also gave K = 4 as the point with the lowest cross-validation error (CV error) (Fig. 4d).

### Marker-trait associations

**Figure 5:**
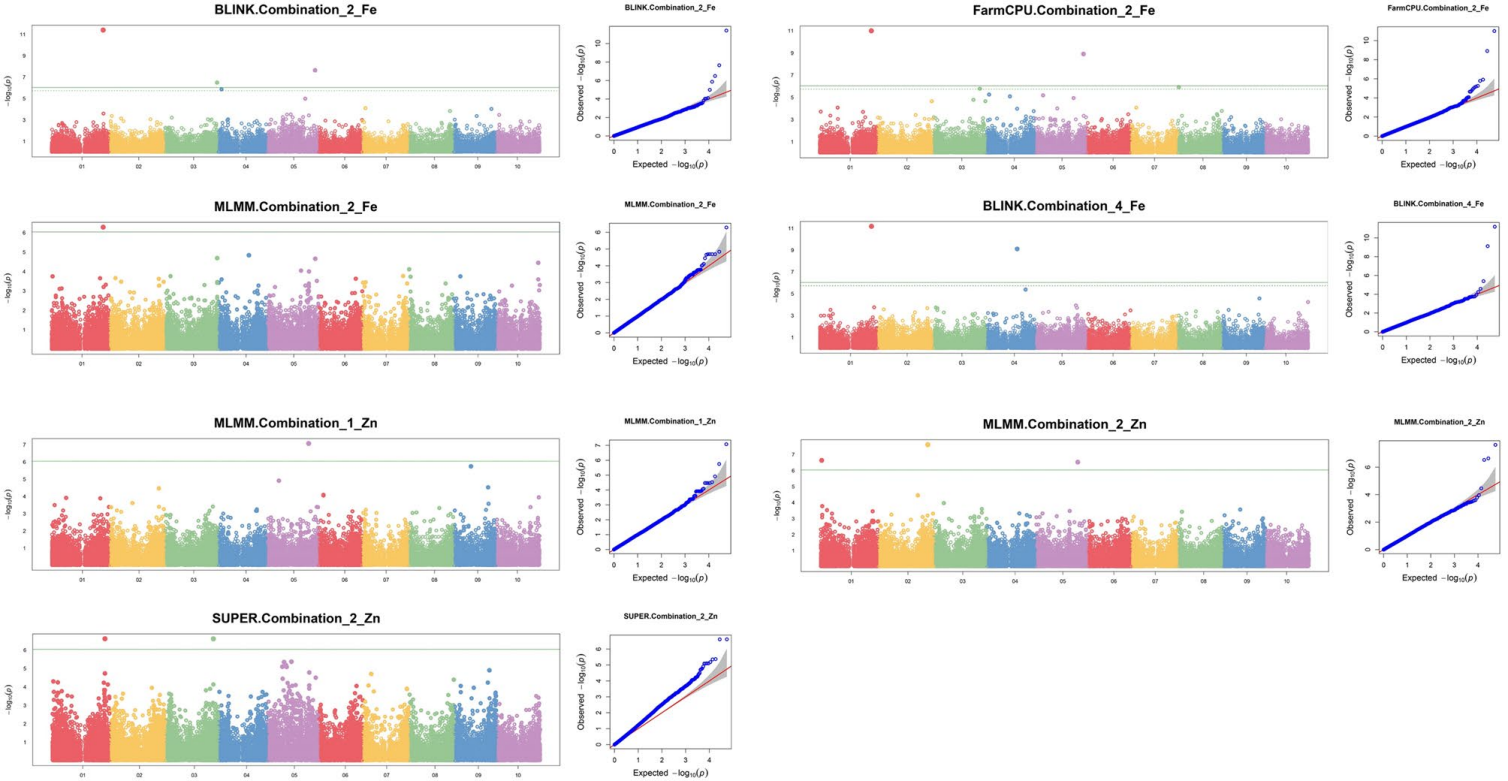
Manhattan plots along with their respective QQ-plots showing the association of sorghum accessions for grain Fe and Zn content. Manhattan (left) and respective QQ-plots (right) (from top to bottom) depicted for grain Fe using BLINK, FarmCPU and MLMM in combination 2; and combination 4 using BLINK; and for grain Zn combination 1 using MLMM; and combination 2 using MLMM and SUPER. Associations were detected using 55,068 high-quality SNPs. The green horizontal line in the Manhattan plot shows the Bonferroni threshold at a 5% level: ${-log}_{10}\left(p.value\right)$ < 6.04, above which solid dots indicated significant MTAs.

A genome-wide association analysis was undertaken to use the whole-genome high-quality 55,068 marker information, high-repeatability phenotypic data, and four single-locus (SUPER) and multi-locus (BLINK, MLMM, FarmCPU) algorithms, and evaluate the linkage disequilibrium (MTAs) that existed between genetic variations and nutritional traits viz., grain Fe and Zn concentration in the panel of 140 sorghum genotypes. For both Fe and Zn traits, five MTAs were detected, each, by BLINK and MLMM GWAS models, whereas FarmCPU and SUPER, each, detected two. A threshold of MAF (≥ 0.05) was set to account for the limitations of GWAS^66^ and to correct for spurious MTAs. Table 3 displays a comprehensive compilation of significant markers that showed significant effects. The Manhattan plots (Fig. 5) showcased the GWAS output, portraying where these highly promising markers assert their statistical significance amidst the vast complexity of the genome. Additionally, these markers were carefully selected based on their elevated values in the Quantile-Quantile (QQ) plots (Fig. 5), indicating their non-random association with the traits of interest.

**Table 3:** The marker-trait associations (MTAs) or SNPs detected for grain iron (Fe) and grain zinc (Zn) using multiple model algorithms with MAF ≥ 0.05.

Traits	SNP	*P* values				PVE (%)	Trial combinations
		MLMM	BLINK	SUPER	FarmCPU		
Fe	S01_72265728	5.19E−07 (2)*	3.88 E−12 (2); 6.47 E−12 (4)	N/A	9.94E−12 (2)	12.0–34.7	2,4
	S03_73164578	N/A	3.20E−07 (2)	N/A	N/A	8.8	2
	S04_43148417	N/A	7.67E−10 (4)	N/A	N/A	8.8	4
	S05_67287071	N/A	2.22E−08 (2)	N/A	1.22E−09 (2)	7.5; 14.7	2
Zn	S01_1495697	2.29E−07 (2)	N/A	N/A	N/A	13.8	2
	S01_73777110	N/A	N/A	2.44E−07 (2)	N/A	12.2	2
	S02_69057239	2.40E−08 (2)	N/A	N/A	N/A	16.5	2
	S03_67027187	N/A	N/A	2.46E−07 (2)	N/A	12.2	2
	S05_58213541	8.40E−08 (1); 2.96E−07 (2)	N/A	N/A	N/A	31.8 (1); 11.4 (2)	1,2

*Numbers within round brackets refer to respective trial combinations in the rightmost (8th) column.

We identified 5 consistent MTAs for Zn and 4 for Fe that were revealed by at least one model in at least one trial combination (Table 3; Fig. 5). Three of the 5 MTAs for Zn were detected using the statistically stringent MLMM model^54,67^ while the remaining 2 were detected using the SUPER model. All Zn MTAs were detected for trial combination 2, except one (S05_58213541), that was detected for trial combinations 1 and 2. The highest PVE reported for Zn was 31.8% (Table 3). The strongest MTA (S01_72265728) reported for Fe was detected by 3 models including the most stringent, MLMM (Table 3). The same MTA was also detected across trial combinations 2 and 4, and was just 1.5 Mb downstream to a Zn MTA locus detected by marker S01_73777110 (Table 3). The highest PVE for this Fe MTA was 34.7%. The other MTAs for Fe were detected by BLINK (S03_73164578, S04_43148417, S05_67287071) and FarmCPU (S05_67287071), all of which had the highest PVE at 14.7%. Supplementary Table S3 depicts further information about significant MTAs along with MAF, effects, genes, and the functional annotation.

### Candidate genes associated with grain Fe and Zn

Candidate genes for each of the MTAs were identified within the window of LD block (51.77 kb upstream–downstream) as defined by the LD decay estimation (Supplementary Table S4; Fig. 4a). Moreover, Table 4 provides a summary of all significant markers that were identified to be localized within genes, and the corresponding annotations. A total of 5 genes were identified for both traits studied (Table 4). Two significant and genic SNPs associated with Fe were linked with Sobic.001G445900 and Sobic.005G188300 genes which play a significant role in Cytochrome P450, heme binding, iron ion binding, oxidoreductase activity, and monooxygenase activity. The genes are putatively involved in other processes including acting on paired donors with incorporation or reduction of molecular oxygen in dhurrin biosynthetic process, jasmonic acid mediated signaling pathway, leaf shaping response to brassinosteroid sterol, and metabolic process unidimensional cell growth. Similarly, three significant SNPs associated with Zn were located in Sobic.001G017500, Sobic.001G463800 and Sobic.003G350800 genes which are putatively associated several properties including NAD(P)-binding domain, Glucose/ribitol dehydrogenase, NADPH-cytochrome P450 reductase, peptidase S8/S53 domain-containing protein and malate dehydrogenase acting in deoxyribonucleoside triphosphate catabolic process, nucleoside triphosphate catabolic process, nucleotide metabolic process, NADH metabolic process, carbon fixation, photosynthesis, magnesium ion binding and metal ion binding.

**Table 4:** A summary of SNP markers located within a gene and the corresponding annotation.

Traits	SNP	Gene ID	Gene position	Sequence description	GeneOntologyID	GeneOntology	GO Biological process	GO Cellular component	GO Molecular function
Fe	S01_72265728	Sobic.001G445900.v3.2	72260202–72266394	similar to Cytochrome P450 heme binding iron ion binding oxidoreductase activity	GO:0005783 GO:0080132 GO:0020037 GO:0005506 GO:0004497 GO:0016491 GO:0016705 GO:0010012 GO:0016132 GO:0010268 GO:0009867 GO:0010358 GO:0009741 GO:0016125 GO:0009826	Endoplasmic reticulum fatty acid alpha-hydroxylase activity, heme binding, iron ion binding, monooxygenase activity, oxidoreductase activity, acting on paired donors, with incorporation or reduction of molecular oxygen steroid, 22-alpha hydroxylase activity, brassinosteroid biosynthetic process, brassinosteroid homeostasis, jasmonic acid mediated signaling pathway, leaf shaping response to brassinosteroid sterol, metabolic process unidimensional cell growth	Dhurrin biosynthetic process	Endoplasmic reticulum membrane, cytosol	Heme binding, iron ion binding, monooxygenase activity, oxidoreductase activity, acting on paired donors, with incorporation or reduction of molecular oxygen
	S05_67287071	Sobic.005G188300.v3.2	67285702–67291437	Similar to Expressed Protein	N/A	N/A	N/A	N/A	N/A
Zn	S01_1495697	Sobic.001G017500.v3.2	1495068–1497208	Similar to Putative uncharacterized protein NAD(P)-binding domain Glucose/ribitol dehydrogenase	GO:0000166 GO:0008152 GO:0016114 GO:0016491 GO:0055114	NADPH-cytochrome P450 reductase, Inosine triphosphate pyrophosphatase, Granule-bound starch synthase 1, chloroplastic/amyloplastic	Deoxyribonucleoside triphosphate catabolic process, nucleoside triphosphate catabolic process, nucleotide metabolic process, NADH metabolic process, NADP metabolic process	Cytosol, endoplasmic reticulum membrane, cytoplasm, cytoskeleton, amyloplast	Flavin adenine dinucleotide binding, FMN binding, NADP binding, NADPH-hemoprotein reductase activity, metal ion binding, ATP binding, hydrolase activity
	S01_73777110	Sobic.001G463800.v3.2	73772286–73782185	Similar to Expressed Protein Protein of unknown function DUF566	GO:0005737 GO:0005880 GO:0008017 GO:0051225	Malate dehydrogenase [NADP] 1, chloroplastic, Cyanohydrin beta-glucosyltransferase, Phosphoenolpyruvate carboxylase 3	Malate metabolic process, dhurrin biosynthetic process, carbon fixation, photosynthesis, tricarboxylic acid cycle	Cytoplasm, endoplasmic reticulum membrane	Malate dehydrogenase (NADP +) activity, cyanohydrin beta-glucosyltransferase activity, magnesium ion binding, iron ion binding
	S03_67027187	Sobic.003G350800.v3.2	67025951–67028277	Expressed Protein HSP20-like chaperone	GO:0042538 GO:0009941	Peptidase S8/S53 domain-containing protein,	Hyperosmotic salinity response, proteolysis, chloroplast organization, heat acclimation	Golgi apparatus, membrane, cytoplasm	Serine-type endopeptidase activity, alpha-mannosidase activity, carbohydrate binding, metal ion binding, RNA cap binding

### Haplotype block analysis

**Figure 6:**
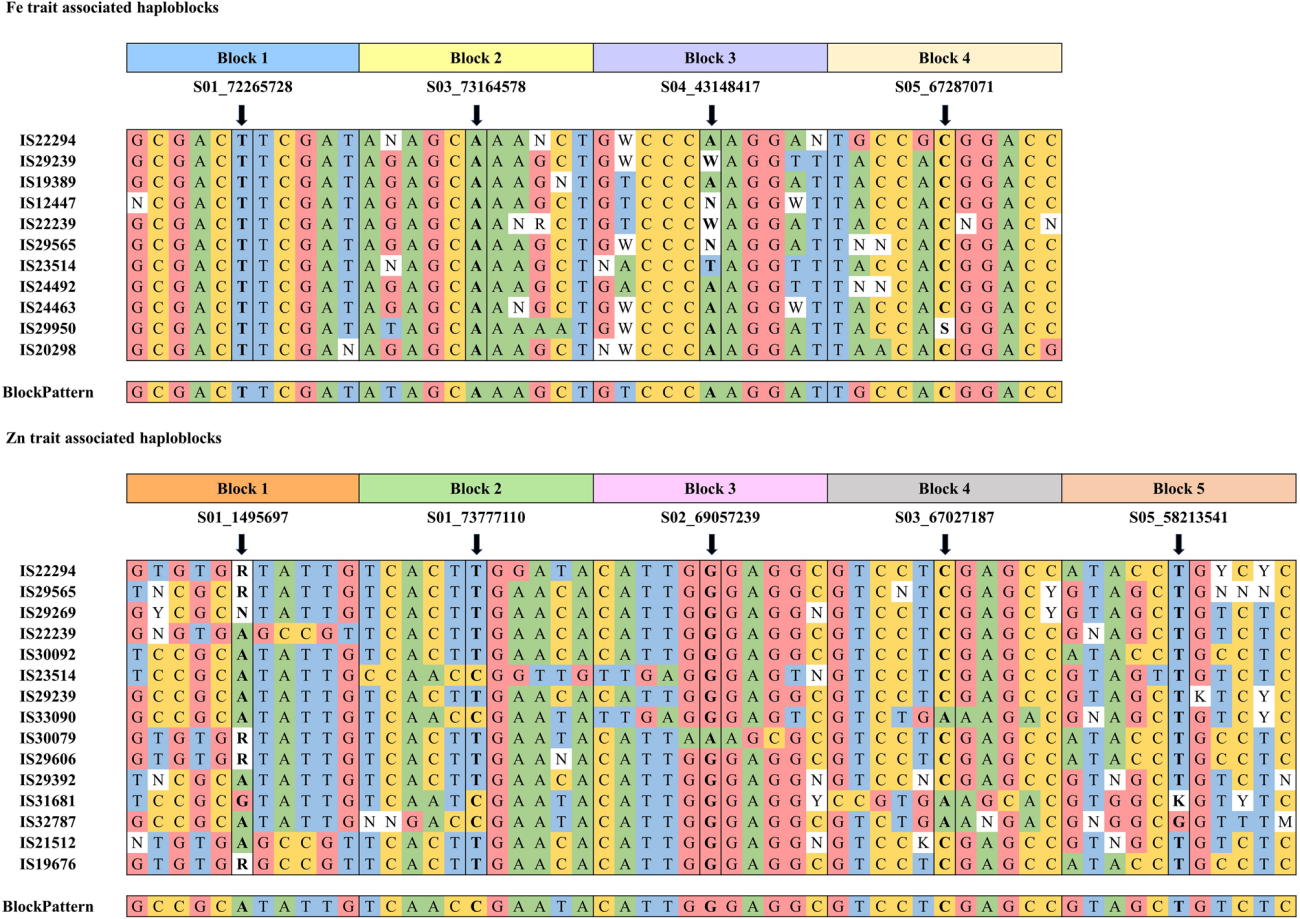
Haplotype blocks for the significant MTAs for traits studied. Five adjacent markers on both sides of the significant SNP were used to find the significant haplotype block. The ‘BlockPattern’ at the end of the haplotype blocks describes the pattern of the respective hapblock.

In the analysis of trait-associated markers, an adjacent set of five markers was utilized to assess the haplotype block for each marker. This approach resulted in an average block size of 109 kb for iron (Fe) trait markers and 66 kb for zinc (Zn) trait markers. The analysis identified a total of eleven accessions exhibiting promising characteristics for Fe traits, and fifteen accessions demonstrating favorable attributes for Zn traits. Notably, five accessions viz., IS22294, IS29239, IS22239, IS29565, and IS23514 were found to possess haplotypes blocks of markers associated with both Fe and Zn traits (Fig. 6). Additionally, based on the Zn haploblock analysis, three accessions (IS22239, IS21512, and IS19676) demonstrated unique pattern of haplotypes indicating that these are related and have the strong variation in Block 1.

### Expression analysis

**Figure 7:**
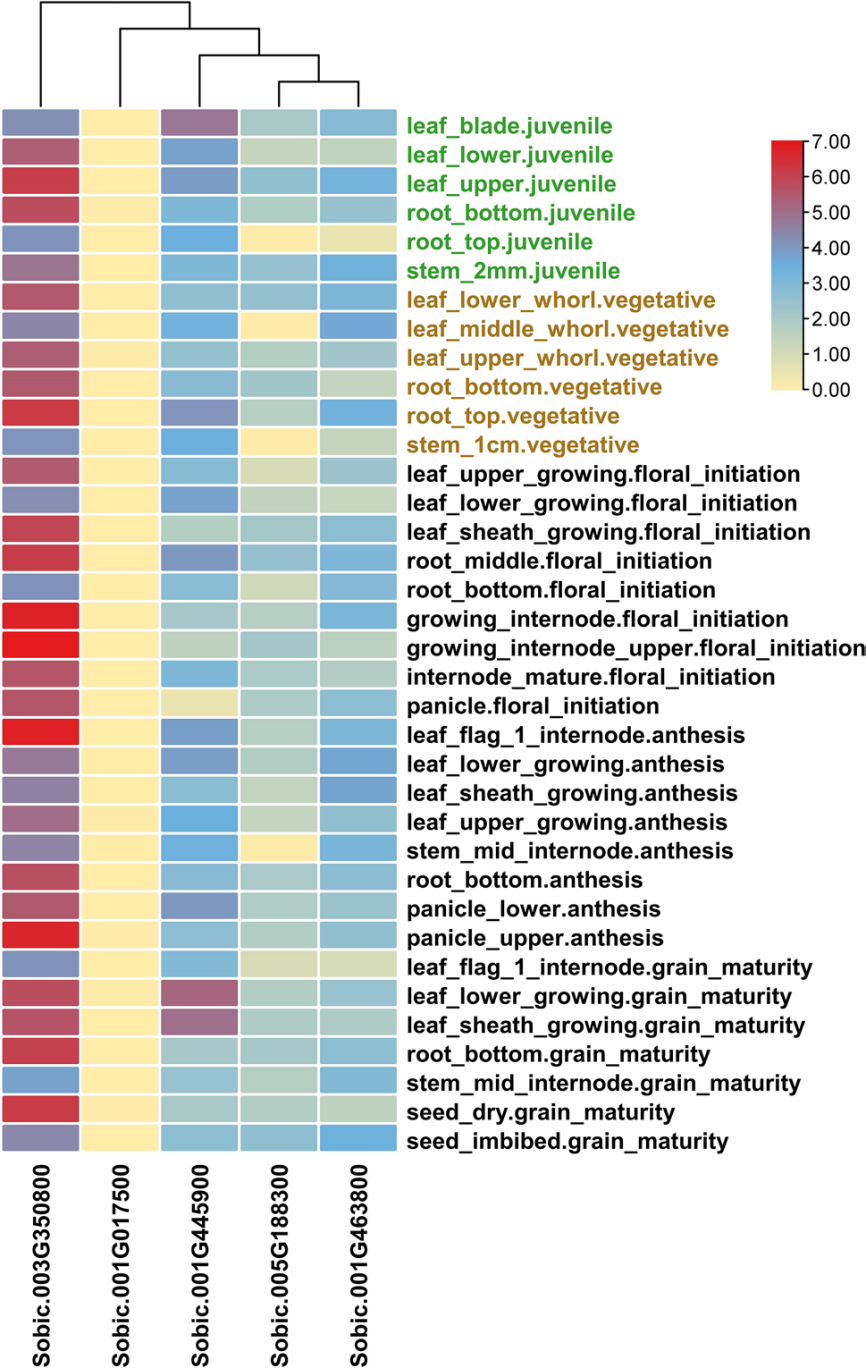
Tissue-specific expression of candidate genes identified for Fe and Zn contents. The expression of five candidate genes in 36 tissues from three different growth stages (juvenile, vegetative, and reproductive) are plotted in the heatmap. Juvenile, vegetative, and reproductive stages are depicted in green, brown, and black font color, respectively.

The tissue-specific expression of the identified genes was analyzed in 36 sorghum tissues at three different growth stages of the plant (Supplementary Table S5, Fig. 7). Based on the FPKM values, an expression heatmap was generated, plotting the expression data of five candidate genes across 36 sorghum tissues (Supplementary Table S5, Fig. 7). Out of five genes, Sobic.003G350800 which is a peptidase S8/S53 domain-containing protein, showed higher level of expression in several tissues such as leaf, root, flower, panicle, and stem. Conversely, Sobic.001G017500 is not expressed in any of the tissues. Sobic.005G188300 and Sobic.001G463800 genes are expressed moderately at grain maturity and anthesis stages in leaf, root, panicle, and seed tissues. Sobic.001G445900 gene showed high expression at juvenile and grain maturity stage.

## Discussion

Iron (Fe) and zinc (Zn) are essential micronutrients for both human and animal nutrition, and their importance in sorghum lies in their role in promoting overall health and preventing nutrient deficiencies, also known as hidden hunger. In addition to preventing micronutrient malnutrition, these micronutrients are essential for cognitive development, immune system function, agricultural productivity, and socio-economic development. Hence, biofortified sorghum provides a readily available and affordable source of essential micronutrients, preventing and alleviating micronutrient deficiencies and preserving traditional food culture. It preserves traditional food preferences, promotes economic development, and contributes to climate resilience as sorghum is a C4 crop with high level of resource use efficiency. Few biofortified sorghum varieties were released under the world’s drylands and this gap motivated our research work; we aimed to identify the genomic regions associated with grain Fe and Zn for downstream use in sorghum biofortification breeding. Several GWAS studies has identified the genes governing complex traits, revolutionizing agricultural improvement. Research on pulses and grains revealed genes related to the accumulation of micronutrients, including the pathway for the carotenoid production in maize^68^. Additionally, wheat displayed 92 SNP trait correlations linked to 10-grain mineral areas. Twenty genes were functionally annotated to demonstrate their significance in grain mineral accumulation; most of these genes were found in the D-genome, indicating control over wheat grain mineral diversity^69^.

The genetic architecture of seed molybdenum and selenium in wild and cultivated chickpea was investigated using GWAS^70^. After 180 entries were surveyed, 16 SNPs were linked to these characteristics. Similarly, 22 quantitative trait nucleotides (QTNs) for grain nitrogen were found by phenotyping 174 accessions of Croatian common bean land races for the seed concentrations of eight micronutrients^71^. In our study, using sorghum entries from the ICRISAT’s minicore provided a guarantee that the uncovered major QTLs and proxy SNPs would benefit wider research communities. To the best of our knowledge, this work is the first to report on the marker traits associations for grain Fe and Zn in sorghum using SNPs and minicore lines.

In this study, the analysis of variance (ANOVA) was carried out for both the characters viz. grain Fe and grain Zn over four combinations of environments. The result indicates highly significant differences among the genotypes for both traits, which revealed the existence of sufficient variation for effective GWAS, statistical inferences, and selection of superior plant ideotypes. Despite the study's small population size, the identified MTAs were significant and agreed with previous results, indicating that the population size did not pose severe data quality challenges^33,72,73^. This study demonstrated that GWAS is a powerful tool for identifying potential genetic factors that contribute to important traits in sorghum genotypes, even with a small sample size.

The repeatability for different combinations was varied, the highest was measured for Fe and Zn (56% and 74%, respectively) in combination 4, whereas low values were registered in combination 2 i.e., 41% and 57% for Fe and Zn, respectively. The genetic basis of traits like grain Fe and Zn content can be complex, involving multiple genes with small effects^24^, and this can explain the observed moderate repeatability. The observed medium to high broad-sense heritability/repeatability and wide variation within population for the evaluated micronutrients are the precondition for a successful GWAS^74–76^. The association between grain Fe and Zn showed significant and high positive values and the trend was similar across environments, implying that selection for either mineral can be used as proxy for the other. Such a favorable relationship between these micronutrients were reported earlier in sorghum^77–80^ and other cereals, such as pearl millet^81–86^, maize^87,88^, rice^89,90^, and wheat^91–94^.

Research on sorghum grain Fe and Zn so far could only identify a few small effect QTLs and some putative candidate genes^24^, without meaningful use in sorghum genetic biofortification. In this study we used multiple popular GWAS models i.e., MLMM, BLINK, FarmCPU and SUPER^33,37^; the latter is a single locus model, while the remaining three are multi-locus. The use of multiple models allowed to detect more reliable QTLs i.e., those that were co-detected by more than one model^95^. On the other hand, although multi-locus GWAS models showed advantage over single-locus GWAS methods^96^, a combination of single-locus methods and multi-locus methods was used in this work as recommended by^97–100^ to improve the detection power and robustness of GWAS. The FarmCPU method offers superior statistical power by dividing Multiple Loci Linear Mixed Models into fixed effects model (FEM) and random effects model (REM), removing confounding, and controlling false positives; the SUPER model addresses computing issues with MLM, while BLINK improves statistical power with using LD information^33,54^. Structure analyses showed that the minicore population was genetically structured with an estimated four subpopulations, and corrective measures were implemented in the GWAS to account for population structure and cryptic relationships to avoid false positive associations^33,101,102^.

With the use of GBS SNP data, we identified nine highly significant MTAs for grain Fe and Zn with P values ranging from 3.88 × 10^–12^ to 5.19 × 10^–7^, explaining 7.5 to 34.7% of phenotypic variation (PVE). Four MTAs for Fe were identified on chromosomes 01, 03, 04 and 05 while five MTAs for Zn were identified on chromosomes 01, 02, 03 and 05. Two major SNPs were identified for Fe and Zn: for iron, the SNP S01_72265728 was identified in the cytochrome P450 gene, while the SNP S05_58213541 associated with Zn is intergenic and near Sobic.005G134800 (2.8 kb) which codes for a zinc binding ribosomal protein. S01_72265728 was associated with positive effect on Fe accumulation in the kernel, while S05_58213541 was associated with negative effect on Zn accumulation. Many other differentially expressed genes are involved in the uptake of minerals. In the case of the gene for Fe, the Cytochrome P450 gene is involved in mineral uptake for both Fe and Zn. Cytochrome P450 is a pigment with heme-protein properties and participates in several catalytic processes that involve specific heme group^103^. Cytochrome P450s catalyze a wide range of chemical reactions and have different enzymatic mechanisms and complex substrate specificities. The enzyme structures of different P450 possess a heme-binding mode with an unusually long heme-binding loop and a unique I-helix which may involved in Fe uptake from the soil^104^. A study on barley collection identified single-nucleotide polymorphisms for Fe and Zn biofortification in which cytochrome P450 superfamily protein was found to be involved in element transport, iron, and zinc binding^43^. Plant cytochrome P450 (P450) participates in a wide range of biosynthetic reactions and targets a variety of biological molecules^105^. However, there is no specific information on how cytochrome P450 gene is involved in Fe and Zn uptake. Satyavathi et al.^106^ identified a cytochrome P450 superfamily protein involved in element transport, iron, and zinc binding, and found out that it was present in the genotypes with high Fe and Zn contents. A study on rice biofortification with zinc and selenium found that the expression pattern of a Cytochrome P450 (CYP) gene followed the mineral accumulation in flag leaves^107^. Such mixed reports suggests that more research is needed to fully understand the function of cytochrome P450 gene in mineral uptake. In pearl millet, it was reported that the cytochrome P450 proteins are up-regulated during panicle initiation^105^. The process of iron uptake by plants is an extremely energy-intensive mechanism^108^. A plant's ability to extract iron from the complex or chelating molecule by reducing Fe+++ to Fe +  + is essential for iron absorption^108^. Uptake of Fe from the soil is dependent on other cations in the soil solution such as manganese (Mn) and calcium (Ca). Accumulation of Fe in grain is a very complex process and many factors such as polyphenol content and other stresses play a significant role in Fe concentration in the grain. In another study found that drought stress alters iron accumulation in sorghum seeds, and photosynthesis impaired by drought stress might trigger a disturbance in iron homeostasis^109^. The same authors hypothesized the that increased vacuolar transporters and ferritin might be involved in the regulation of iron accumulation in sorghum seeds under drought stress.

Zinc-binding proteins are involved in abiotic stress tolerance and play a significant role in root hair growth under stress conditions. It was reported that the metal binding proteins facilitates the absorption of Zn and metal ions by procuring the binding sites^110^ but in our study, the identified SNP (S05_58213541) showed a negative effect which could mean that there is an association between that SNP and a particular trait, but the presence of a specific allele at that SNP locus is associated with a decrease or reduction in the trait of interest, Zn concentration in this case. This can indicate that either the plant absorbed the Zn ions from the soil and sequester it but unable to translocate it to the grains, or the plant with that particular SNP was unable to uptake enough Zn form the soil. Whether this is a direct effect (modified gene product) or a non-allelic interaction, it is to be investigated. This gene can also be a candidate for genome editing in order to improve Zn concentration in the kernel. If the associated gene is knocked out, there may be chances of significantly increasing the concentration of Zn in grains. Most putative genes identified in this study are zinc-binding or zinc ion-binding proteins. The gene ontology CCHH term indicates the presence of transcripts involved in metal ion binding activity, indicating their role in uptake and transport of Fe and Zn. A zinc finger is a small protein structural motif characterized by the coordination of one or more zinc ions (Zn2 +) to stabilize the fold. Zinc finger proteins are transcription factors with the finger domain, which plays a significant role in gene regulation and are required for transcriptional activation.

Based on the results, among the four combinations of locations, combination 2 consisting of three locations identified the most MTAs. The identified candidate genomic regions/candidate genes are likely to have an important role in achieving high Fe and Zn content in sorghum grains. The identified SNPs can therefore be validated and used in developing nutritionally improved, Fe and Zn rich sorghum cultivars, which would help address micronutrient deficiencies and increase food security. The correlation between Fe and Zn concentration in the sorghum grain was high (r > 0.80) but we could not come across pleiotropic QTLs (SNPs) in this study. The lack of common SNPs in this study may indicates that the effects of the genetic factors that influence Fe and Zn concentrations may vary depending on the environmental conditions. Indeed, a correlation of 0.80 implies that 36% of the variance in sorghum grain Fe or Zn concentration cannot be explained by neither of the two metals.

Haplotype-based breeding has recently come to prominence as an effective approach to developing crop varieties that meet particular requirements^111^. To be used in breeding programs, this breeding strategy needs to first determine superior haplotypes. Haplotypes are distinct sets of alleles found on a single chromosome that are inherited together with a limited probability of contemporary recombination^112^. Breeders can increase the accuracy of genomic predictions and breeding strategies by more effectively defining haplotypes using linkage disequilibrium-based techniques and haplotype diversity^113,114^.

Recently, researchers explained the genetic diversity and evolutionary background of sorghum accessions by identifying several haplotypes of genes such as *Dry*^115^ and *Sh1*^116^ in domesticated and wild sorghum lines. Furthermore, Wu et al.^117^ conducted a population genetic study that demonstrated the *Sh1* and *SbTB1* regions were subject to strong selection during the domestication of sorghum. Moreover, it has resulted in the potential role of *SbTB1* haplotype in controlling the number of lateral branches in sorghum in domesticated and wild accessions. To the best of our knowledge, there is no specific study that directly addresses haplotype analysis in grain Fe and Zn in sorghum, and this is the first study to report haplotypes for grain Fe and Zn. In the present investigation, the haplotype analysis used sets of five markers to evaluate haplotype blocks, resulting in average block sizes of 109 kb for iron (Fe) trait markers and 66 kb for zinc (Zn) trait markers. It identified eleven accessions with promising Fe traits and fifteen with favorable Zn traits. The identified haploblocks were further used to identify sorghum accessions that inherited iron and/or zinc QTLs stably and possibly with rare crossing-over events. Notably, five accessions possessed haplotype blocks associated with both Fe and Zn traits. Furthermore, three accessions showed unique haplotype patterns related to strong variation in Block 1 based on Zn haploblock analysis. Thus, it will be beneficial to: (1) target the identified haploblock-containing plants as potential QTL donors in sorghum crossing blocks, and (2) tailor plant architecture and enhance grain nutrient content by incorporating these haplotypes through haplotype-based breeding in sorghum breeding programs.

In this work, the baseline expression profiles of the identified genes were shown using heat map. The gene Sobic.003G350800 encoding for a protein which contains the peptidase S8/S53 domain, was found to be highly expressed in a variety of tissues, including the leaf, root, flower, panicle, and stem. Nearly all plant species include peptidase S8/S53 domain subtilases, which are subtilisin-like proteases that regulate a variety of biotic and abiotic stressors, and are expressed in a variety of tissues. *Sorghum bicolor* contains 57 genes belonging to the S8 family^118^. Subtilase family members exhibit remarkable functional versatility, from protein expression to stress responses, and modulating plant growth and development from seed development to senescence^119^. Genes, Sobic.005G188300 and Sobic.001G463800 are expressed moderately at grain maturity and anthesis stages in leaf, root, panicle, and seed tissues. These genes mainly encode for malate dehydrogenase (MDH: EC 1.1.1.82) in C4 plants. The functional annotation of Sobic.001G463800 showed its role in iron ion binding activity, which is probably one of the factors determining the success of biofortification, nutrient bioavailability at different plant growth stages, from soil to plant tissues. In nature, the synthesis of malate is catalyzed by the MDH enzyme through the reversible reduction of oxaloacetate to malate. As such, MDH is therefore indirectly but strongly associated with iron and other nutrients uptake in plants. Indeed, Malate is a key product of plant metabolism with diverse functional roles in plants^120,121^, including, but not limited to: respiration and energy generation, photosynthesis (both C3 and C4), fatty acid oxidation, lignin biosynthesis, pulvinal and stomatal function, nitrogen (N2) fixation and amino acid biosynthesis, ion balance, uptake of phosphorus (P) and iron (Fe), and aluminum (Al) tolerance^121–123^. Cultivar differences, such as low nutrient mobility and remobilization efficiency, also affect the effectiveness of biofortification, particularly in relation to leaves and edible parts^124^. The identified candidate genes showing high micronutrients expression in leaves, stem, and grains at different growth stages (juvenile, vegetative, and reproductive stages) can be targeted to increase the nutritional value of both grain and stover, providing health-promoting food, feed, and forage. Overall, this study provides invaluable information on the genetic basis of grain Fe and Zn contents in sorghum and identifies candidate genes and genomic regions that can be used in breeding programs to improve these micronutrients. However, more research works are needed to further characterize these genomic regions and candidate genes and to validate their role in grain Fe and Zn concentration in sorghum grain before they are implemented in sorghum breeding programs.

### Supplementary Information

Supplementary Information.

## Data Availability

The datasets created during and/or analyzed during the current investigation are not publicly available, although the corresponding author can provide them upon justifiable request.
